# Joint awareness in posttraumatic osteoarthritis of the knee: validation of the forgotten joint score in long term condition after tibial plateau fracture

**DOI:** 10.1186/s12955-017-0801-y

**Published:** 2017-12-02

**Authors:** Florian Baumann, Johannes Weber, Daniel Mahr, Martin Bäumlein, Maximilian Kerschbaum, Karolina Müller, Paavo Rillmann, Michael Nerlich, Markus Loibl

**Affiliations:** 1 Department of Trauma Surgery, Regensburg University Medical Center, Regensburg, Germany; 2 Center for Orthopaedics and Trauma Surgery; University Hospital Giessen and Marburg, Marburg, Germany; 3 Center for Clinical Studies, Regensburg University Medical Center, Regensburg, Germany; 4 Spital Davos, Department of Orthopedic Trauma Surgery, Davos, Switzerland

**Keywords:** Patient reported outcome measurement (PROM), Forgotten joint score (FJS), COSMIN checklist, Early osteoarthritis, Tibial plateau fracture

## Abstract

**Background:**

Evaluating patient-reported outcomes (PRO) in early osteoarthritis (OA) of the knee is difficult. Established measurement tools are focused on one of the two major patient groups in knee surgery: young, highly active patients, or older patients with advanced degenerative OA of the knee. Joint awareness in everyday life is a crucial criterion in measuring PRO. The purpose of this study was to validate a German version of the “Forgotten Joint Score” (FJS) in patients after surgical treatment of tibial plateau fractures.

**Methods:**

In this prospective cohort study, clinical and radiological outcomes data were collected from patients after surgical treatment of tibial plateau fractures following a skiing accident. Functional outcome questionnaires were administered including the FJS, the Lysholm-Score, the Tegner-Activity Scale (TAS), the EuroQol-5D (EQ 5-D), and a subjective rating of change. The validation study was carried out according to the COSMIN checklist protocol. The KLS was used to measure the presence and severity of OA on knee radiographs, and correlation with the FJS was measured.

**Results:**

Cronbach‘s alpha was .96 (95%-CI .92, .99) confirming good internal consistency. Test-retest reliability of the FJS was high with an ICC_(67)_ = .91 (95%-CI .85,.95). Furthermore, no relevant floor or ceiling effects were observed. FJS significantly differed in patients with different OA degrees (*p* = .041). Symptomatic patients had significant lower FJS than asymptomatic patients (*p* < .001).

**Conclusions:**

This is the first study validating a disease-specific PRO, the FJS, in long-term outcomes after joint fracture. We demonstrated good psychometric properties and a significant correlation between the FJS and the radiologic degree of OA in patients with a history of tibial plateau fracture.

**Trial registration:**

Clinical Trial Registry University of Regensburg Z-2015-0872-2. Registered 01. October 2015.

## Introduction

Evaluation of patient-reported outcome (PRO) in early osteoarthritis (OA) of the knee is difficult [1–4], with a large range of severity measurements and a variety of symptomatic criteria that patients present with [5, 6]. However, objective measurement of quality of life in mild or moderate OA is of growing interest and can play an important role in the development of joint preservation therapy [5, 7, 8]. Conventional scoring systems are based on objective parameters like the range of motion or radiographic factors. However, this reflects the surgeon’s point of view.

The “Forgotten Joint Score” (FJS) was originally developed as a measurement tool in patients after arthroplasty of the hip or knee joint [9]. Joint awareness in everyday life is a crucial criterion in the activity of daily living FJS [10]. Considering the patient’s evaluation of the loss of awareness of the knee joint is a paradigm shift in PRO measurement [9, 11–14] relative to more traditional measurements of pain or activity level. Conventional scoring instruments frequently show ceiling effects leading to limited content validity [14]. For evaluation of further therapeutic improvements, it will be necessary to discern between good and excellent results. Reflecting the patient’s joint awareness, the FJS has shown high discriminative power in patients after arthroplasty of the hip and knee [15]. Therefore, the interpretation of the patient’s joint awareness measured by the FJS is seen as a new dimension in PRO-measurement.

Established measurement tools focus on one of the two major patient groups in knee surgery: First, young and physically active patients sustaining sports injuries without any signs of OA [16–19], and second, older patients with advanced OA of the knee designated for knee arthroplasty [20–22]. Recently, a study by Behrend et al. [23] demonstrated that the FJS is a viable instrument for PRO measurement in patients after anterior cruciate ligament (ACL) reconstruction. The FJS could serve as an ideal PRO measurement for other sports-related knee injuries resulting in increased risk of developing OA. Accordingly, the FJS could become an invaluable measurement tool in evaluating long-term outcomes in patients sustaining tibial plateau fractures, who are predisposed to posttraumatic OA of the knee joint. In this study, we intended to investigate the relationship between the FJS and mild to moderate posttraumatic OA at long-term follow-up. For this reason, we chose to validate the score in a specific patient population. A group of patients after knee joint fracture with long-term follow-up seemed to be feasible in this context.

The purpose of this study was to validate a German version of the “Forgotten Joint Score” (FJS) according to the COSMIN (COnsensus based Standards for the selection of health status Measurement INstruments) checklist. For determination of construct validity, we investigated the correlation between the FJS and long-term radiographic development of OA as measured by the Kellgren-Lawrence score (KLS) in patients after surgical treatment of tibial plateau fractures following a skiing accident.

### Materials and methods

The COSMIN checklist (COnsensus based Standards for the selection of health status Measurement INstruments) is a consensus-based checklist to evaluate the methodological quality of studies on measurement properties of health status measurement instruments based on an international Delphi study in 2010 [24]. The COSMIN checklist was utilized in this study to ensure high methodological quality [25]. This study was carried out in accordance with the Declaration of Helsinki and approved by the ethics committee at the University of Regensburg in December 2015 (Institutional Review Board Number 15–101-0241). We obtained written informed consent from all study participants.

### Study design

We identified 108 consecutive German-speaking patients who sustained an intraarticular tibial plateau fracture in a skier’s accident between 03/2000 and 12/2006 (T0).

Inclusion criteria were:

1. Patients with history of undergoing open reduction and internal fixation (ORIF) of an intra-articular tibial plateau fracture
2. No relevant concomitant injuries,
3. No preexisting mental disorder,
4. Minimum follow up was 8 years past trauma,
5. Age between 18 and 70 years,
6. Minimum light sports activity level (Tegner Activity Scale ≥3) at time of injury,
7. Sufficient German reading and comprehension capacity, and
8. Consent to participate in this study.

77 patients met the inclusion criteria. For characterization of the patient population, we recorded relevant clinical data and reviewed pre- and initial postoperative x-rays (T0). For the validation study (T1 and T2), the patients were asked to answer the following questionnaires according to their current status and return the forms by mail. We reminded all patients who did not answer within two weeks by telephone. For evaluation of test–retest reliability, the patients completed a second questionnaire after a minimum of two weeks (T2).

### Materials

### Forgotten joint score knee (FJS)

The FJS is a self-administrated questionnaire comprising of 12 items concerning the patient’s lack of awareness of the knee joint in everyday life [9]. The loss of awareness of a joint is widely regarded as the ultimate goal in achieving maximum patient satisfaction [9]. Developed in 2012, the FJS has shown a high internal consistency, construct validity and responsiveness in long term PRO [9, 11, 12, 15, 23, 26–30]. The FJS has been validated in patients after arthroplasty of the knee or hip, and after ACL reconstruction [23]. The total score ranges from 0 (low degree of forgetting) to 100 (high degree of forgetting).

### Lsyholm knee scoring scale LH [3]

The LH is a well-established 8-item PRO tool to evaluate the functional status of the knee in physically active patients [19]. The score values of each question are summed up to representing the total score ranging from 0 points (representing extreme limitations and worst outcome) to 100 points (representing full function and best outcome). The score has been previously validated in German [31].

#### Tegner activity scale (TAS)

The TAS is a 10 level activity scale reflecting the patient’s currently highest level of sports activity or other routine activities [18]. It was designed to complement other functional scores for the knee joint, and is the most commonly used activity-scoring tool for patients with knee disorders. A German version is available [32].

#### EuroQol-5D 3 L (EQ 5-D)

The EQ-5D is a global quality of life questionnaire consisting of a 5-item assessment of the health status regarding mobility, self-care, usual activities, pain/discomfort, and anxiety/depression combined to an EQ Index ranging from −.21 (low quality of life) to 1.00 (high quality of life) [33]. The second part of the EQ-5D consists of a visual analogue scale (EQ VAS) concerning the patient’s assessment of the current global health status ranging from 0 (worst health status) to 100 (best health status).

#### Subjective assessment

The patient was asked to evaluate at T2 whether the condition of his artificial knee joint was ‘better’, ‘somewhat better’, ‘unchanged’, ‘somewhat worse’ or ‘worse’ compared to T1. This item was used as the anchor variable for test-retest reliability of FJS.

#### Radiologic assessment

Radiologic assessments were based on plain radiographs of the knee in two planes. We evaluated preoperative x-rays, postoperative control x-rays (T0) and radiographs at the time of follow-up (T2). A single experienced independent observer evaluated the degree of degeneration according to the clinical relevant classification of KLS: 1) no OA (KLS = 0), 2) mild OA (KLS = 1 or 2), 3) severe OA (KLS = 3 or 4) [34]. These parameters were rated at three time points and separately for all joint compartments (medial, lateral, and patellofemoral).

### Statistical analysis

Statistical analysis was performed using the software package SPSS (Version 24, SPSS Inc., Chicago, Illinois). The level of significance was defined at *p* < .05 for all tests. Descriptive data are given as frequencies (n) and percentage (%) for categorical variables, means (m) and standard deviations (±) for continuous and normal distributed variables, and median (med) and quartiles (Q1/Q3) for continuous and not normally distributed variables. Normal distribution was assessed by Shapiro-Wilk-Test.

### Methodological testing according to the COSMIN checklist

Studies evaluating measurement properties have to meet a high methodological quality [25]. The COSMIN checklist (COnsensus based Standards for the selection of health status Measurement INstruments) is an international consensus-based checklist to evaluate the methodological quality of health status measurement instruments [24]. Based on the COSMIN checklist, we evaluated the reliability (internal consistency, test-retest reliability) and validity (construct validity, clinical validity, content validity) of the FJS.

Internal consistency is described as the degree of interrelatedness among items [35]. Sufficient internal consistency was assumed for a Cronbach’s α > .70 [25]. Test–retest reliability is the extent to which results of the same patient in the same health condition remain unchanged over time [35]. According to the recommendation of the COSMIN guidelines, the retest was performed after a minimum of two weeks after primary consultation to avoid recollection of the answers and relevant changes in health condition. Intraclass correlation coefficient (ICC) was calculated for all patients indicating an unchanged condition of their knee joint since the primary evaluation. For an ICC > .70 sufficient test-retest reliability was assumed [25].

Since there is no gold standard in the measurement of PRO, validity was rated as construct, clinical and content validity. Construct validity is the degree to which the score of the FJS is consistent with the scores of questionnaires (LH, TAS, EQ Index, EQ VAS) indicating to measure the same construct (congruent validity) [35]. Construct validity was measured by Spearmen’s rank correlation. Correlation coefficients ≥ .40 indicates congruent validity. Clinical validity of FJS was measured by known-groups comparisons: Kruskal-Wallis test was used for differences in OA degrees and U-test was used for differences between symptomatic and asymptomatic patients. Content validity is met by the absence of floor and ceiling effects. If more than 15% of patients score highest (100) (ceiling effect) or lowest (0) value (floor effect) in the FJS, extreme outcome values might not be represented adequately and the questionnaire might not be able to reflect changes [25].

## Results

### Demographic data and generalizability

#### Demographic and clinical data

**Fig. 1:**
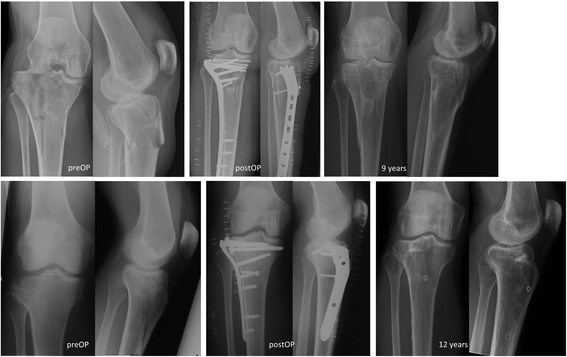
Radiographs showing preoperative, postoperative, and long-term condition after tibial head fracture. Patient 1 nine years after bicondylar tibial head dislocation fracture. Patient 2 sustained a lateral depression type tibial head fracture 12 years ago

77 patients (51% women) after surgical treatment of tibial plateau fractures following a skiing accident were included in the study. All patients were treated operatively at Spital Davos (CH) with open reduction and internal fixation (ORIF) 1.4 days ±1.2 (range 0–6) after the accident. For stabilization, 40% received only compression screw fixation, and 60% received an angular stable plate osteosynthesis with or without additional compression screw fixation. Operative management was carried out according to the AO-principles. The postoperative regimen was equal for all patients with partial weight-bearing for 6 weeks. The median time span between accident (T0) and first FJS assessment (T1) was 13 years (Q1/Q3 = 12/15, range = 9–13). The mean age at T1 was 63.2 ± 12.2 years (range 36–87). Figure 1 shows two example patients 9 and 12 years after a tibial head fracture.

#### Reliability

Cronbach’s alpha of .96 showed high internal consistency for the FJS. The item total correlation ranged between .95 and .96. The ICC_(67)_ was .91 (95%-CI = .85, .95) for all patients indicating an unchanged condition of their knee joint since their primary evaluation (T1). The median time span between first (T1) and second (T2) FJS assessment was 26 days (Q1/Q3 = 24/32, range = 2–113).

#### Validity

There was no floor effect (no Patient had a minimum score of 0) and no relevant ceiling effect (10% (*n* = 8) patient had a maximum score of 100) for the FJS (T1).

Construct validity (T2) could be confirmed between FJS and LH (r_s_ = .71, *p* < .001) as well as between FJS and EQ VAS (r_s_ = .51, p < .001) indicating that these questionnaires /scales measure the same construct. The coefficient of the correlation between FJS and EQ Index (r_s_ = .35, *p* = .002) fell short of reaching the cut-off of ≥40 indicating that the scales are not conceptually related. TAS correlated low, but significant with FJS (r_s_ = .28, *p* = .013). The higher the activity, the higher the forgetting of the joint.

**Fig. 2:**
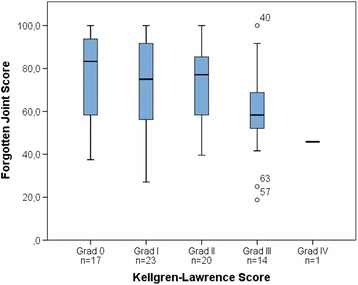
Relation between Kellgren-Lawrence Score (KLS) and Forgotten Joint Score (FJS) at T2

The Kruskal-Wallis test demonstrated significant differences between groups of patients with different degrees of OA in FJS values at T2 (H_(2, 75)_ = 6.370, *p* = .041). Figure 2 shows the relation between KLS and FJS. At T2, asymptomatic patients had significantly higher FJS values (med = 81.3, Q1/Q3 = 62.0/91.7) than symptomatic patients (med = 54.2, Q1/Q3 = 41.7/75.0, *p* < .001).

## Discussion

In this prospective study, we demonstrate that the FJS is a valid and reliable PROM-tool in patients after surgical treatment of tibial plateau fractures following a skiing accident. The FJS correlated with the radiologic degree of joint degeneration at long-term follow-up (Kellgren-Lawrence Score) and was able to distinguish between clinically symptomatic and asymptomatic patients. This is the first study following the complete COSMIN checklist validating FJS in long-term results after joint fracture.

### Study design and patient population

Early OA of the knee joint is defined as knee pain with radiographic changes or arthroscopically visualized cartilage damage [36]. Early OA is a disabling condition with morphologic degenerative changes, however with a certain capacity for compensation/regeneration [5, 8, 37]. Patients with mild to moderate OA can present with a variety of signs and symptoms. Moreover, dynamics of joint degeneration kinetics vary greatly, which makes it difficult to characterize this patient population, and to compile comparable study populations [5, 8, 36]. If OA is the consequence of an acute event, like in posttraumatic OA, a pro-inflammatory response is triggered initially in addition to the osteochondral injury. After the remodeling of damaged cartilage areas, there can be a long period of asymptomatic steady-state in post-traumatic joint disease before further progression of degenerative disease. Only 12% of patients with OA of the knee have a relevant knee injury in their medical history [38]. However, this patient population represents an ideal opportunity to study early indicators of the progression of OA. Hence, we chose to validate the FJS as a long-term PRO after tibial plateau fracture. To minimize confounding, we set our exclusion criteria to ensure a homogenous patient population with a similar level of activity. Our demographic data is comparable to other studies on sports-related tibial plateau fracture with an average age around 50 years [39]. Originally, the FJS was designed for patients after arthroplasty of the hip and knee joint [9]. Thienpont [26] validated the score for patients with advanced OA designated for arthroplasty. Unfortunately, they do not provide data on the radiologic degree of OA preoperatively. Thienpont et al. [26] recorded a mean FJS of 24 points preoperatively indicating significant joint awareness in advanced OA of the knee. Although originally utilized for older patients, the FJS has been shown to be equally reliable in younger patients in recent studies [23, 26]. Behrend et al. [23] recently published validation data of the FJS on mid- and long-term results after ACL-reconstruction in 115 patients, demonstrating an increased joint awareness of 20 points after ACL-reconstruction compared to matched healthy control subjects. Patients after ACL-reconstruction had a mean FJS-value of 71.6 (mid-term) and 70.1 (long-term). These results are comparable to the findings in the present study on long-term outcome after tibial plateau fracture, with a mean FJS of 70 points.

### Reliability

The FJS has been validated in English and has been adapted in French, Dutch, Danish, Japanese, and German language [9, 26–30]. All publications confirmed internal consistency with a Cronbach’s alpha of 0.95–0.97. In the present study, we recorded a Cronbach’s alpha of 0.96. According to Terwee et al. [25], a positive rating for internal consistency can be given if Cronbach’s alpha is between 0.7 and 0.95. Greater values reflect higher correlations among the items and might be an indication for a redundancy of two or more items [25]. Cronbach’s alpha is dependent on the number of items, leading to higher values for scores with a higher number of items. However, the FJS consists of only 12 items. Hence, the concept of the FJS with the inception of awareness for every question might be somewhat prone for a high correlation among the items. Test-retest reliability for the FJS has been documented to be between 0.80 and 0.94 [27, 28, 30]. We could confirm excellent test-retest reliability with an ICC_(68)_ of 0.91. We investigated a long-term result with a minimum follow-up of eight years after injury. Therefore, a stable medical condition can be expected to make the ICC relatively robust.

### Validity

The LH and the TAS seemed most appropriate for evaluation of the construct validity on a functional basis, because they are widely used and validated in German language for sports-related injuries and arthroplasty patients [31–33].

A major issue in outcome measurement of the knee joint is the correlation between clinical and radiologic results. Especially in mild to moderate OA, conventional outcome measurement tools often fail to reflect the radiological status of OA [1, 40]. However, large cohort studies like the ROAD study [41] have shown that there is an impairment of disease-specific and generic health-related quality of life (HRQoL) scales [7, 41]. Considering this, a PRO-measurement tool for early posttraumatic OA should reflect the disease-specific impairment of HRQoL.

The FJS showed good correlation to the Kellgren-Lawrence score in our patient population and was able to distinguish between symptomatic and asymptomatic patients. In addition, we saw significant differences in FJS values between groups of patients with no OA (KLS = 0), mild OA (KLS = 1 or 2), and severe OA (KLS = 3 or 4) indicating good construct validity.

### Limitations

The results of this study should be interpreted in light of some limitations. First, the study design specifically investigates mild to moderate posttraumatic OA making our results less generalizable to primary OA. In addition, we could not control the factor degeneration due to natural aging of the joint or overuse with this study design. Another limitation is that no conservatively treated patients were included as all patients included in the study were managed operatively. However, the majority of intraarticular tibial plateau fractures are treated operatively.

## Conclusion

This is the first study on validation of the FJS as a long-term indicator of progression to mild or moderate of post-traumatic OA after intra-articular joint fracture. We demonstrate good psychometric properties in our patient population and confirm a correlation between the radiologic degree of OA and the disease-specific PRO-score result of the FJS. The FJS was able to distinguish between symptomatic and asymptomatic patients, as well as between mild and severe forms of radiographically diagnosed OA.

## References

[1] Parsons C, Clynes M, Syddall H, Jagannath D, Litwic A, van der Pas S, et al. How well do radiographic, clinical and self-reported diagnoses of knee osteoarthritis agree? Findings from the Hertfordshire cohort study. SpringerPlus 2015;4:177. doi: 10.1186/s40064-015-0949-z. PubMed PMID: 25932366; PubMed Central PMCID: PMC4408304. 10.1186/s40064-015-0949-z PMC4408304 25932366

[2] Ramkumar PN, Harris JD, Noble PC. Patient-reported outcome measures after total knee arthroplasty: a systematic review. Bone & joint research 2015;4(7):120–127. doi: 10.1302/2046-3758.47.2000380. PubMed PMID: 26220999; PubMed Central PMCID: PMC4602194. 10.1302/2046-3758.47.2000380 PMC4602194 26220999

[3] Engelhart L, Nelson L, Lewis S, Mordin M, Demuro-Mercon C, Uddin S, et al. Validation of the knee injury and osteoarthritis outcome score subscales for patients with articular cartilage lesions of the knee. Am J Sports Med 2012;40(10):2264–2272. doi: 10.1177/0363546512457646. PubMed PMID: 22962288. 10.1177/0363546512457646 22962288

[4] Roos EM, Toksvig-Larsen S. Knee injury and osteoarthritis outcome score (KOOS) - validation and comparison to the WOMAC in total knee replacement. Health Qual Life Outcomes 2003;1:17. doi: 10.1186/1477-7525-1-17. PubMed PMID: 12801417; PubMed Central PMCID: PMC161802. 10.1186/1477-7525-1-17 PMC161802 12801417

[5] Madry H, Kon E, Condello V, Peretti GM, Steinwachs M, Seil R, et al. Early osteoarthritis of the knee. Knee surgery, sports traumatology, arthroscopy : official journal of the ESSKA. 2016;24(6):1753–1762. doi: 10.1007/s00167-016-4068-3. PubMed PMID: 27000393. 10.1007/s00167-016-4068-3 27000393

[6] Case R, Thomas E, Clarke E, Peat G. Prodromal symptoms in knee osteoarthritis: a nested case-control study using data from the osteoarthritis initiative. Osteoarthr Cartil 2015;23(7):1083–1089. doi: 10.1016/j.joca.2014.12.026. PubMed PMID: 25843364; PubMed Central PMCID: PMC4491193. 10.1016/j.joca.2014.12.026 PMC4491193 25843364

[7] Kiadaliri AA, Lamm CJ, de Verdier MG, Engstrom G, Turkiewicz A, Lohmander LS, et al. Association of knee pain and different definitions of knee osteoarthritis with health-related quality of life: a population-based cohort study in southern Sweden. Health Qual Life Outcomes 2016;14(1):121. doi: 10.1186/s12955-016-0525-4. PubMed PMID: 27565135; PubMed Central PMCID: PMC5002211. 10.1186/s12955-016-0525-4 PMC5002211 27565135

[8] Anderson DD, Chubinskaya S, Guilak F, Martin JA, Oegema TR, Olson SA, et al. Post-traumatic osteoarthritis: improved understanding and opportunities for early intervention. Journal of orthopaedic research : official publication of the Orthopaedic Research Society 2011;29(6):802–809. doi: 10.1002/jor.21359. PubMed PMID: 21520254; PubMed Central PMCID: PMC3082940. 10.1002/jor.21359 PMC3082940 21520254

[9] Behrend H, Giesinger K, Giesinger JM, Kuster MS. The "forgotten joint" as the ultimate goal in joint arthroplasty: validation of a new patient-reported outcome measure. J Arthroplast 2012;27(3):430–436 e1. doi: 10.1016/j.arth.2011.06.035. PubMed PMID: 22000572. 10.1016/j.arth.2011.06.035 22000572

[10] Filbay SR, Ackerman IN, Russell TG, Macri EM, Crossley KM. Health-related quality of life after anterior cruciate ligament reconstruction: a systematic review. Am J Sports Med. 2014;42(5):1247-55. doi:10.1177/0363546513512774. PubMed PMID: 24318609. 10.1177/0363546513512774 24318609

[11] Giesinger JM, Kuster MS, Behrend H, Giesinger K. Association of psychological status and patient-reported physical outcome measures in joint arthroplasty: a lack of divergent validity. Health Qual Life Outcomes 2013;11:64. doi: 10.1186/1477-7525-11-64. PubMed PMID: 23601140; PubMed Central PMCID: PMC3639925. 10.1186/1477-7525-11-64 PMC3639925 23601140

[12] Thienpont E, Opsomer G, Koninckx A, Houssiau F. Joint awareness in different types of knee arthroplasty evaluated with the forgotten joint score. J Arthroplast 2014;29(1):48–51. doi: 10.1016/j.arth.2013.04.024. PubMed PMID: 23688851. 10.1016/j.arth.2013.04.024 23688851

[13] Eymard F, Charles-Nelson A, Katsahian S, Chevalier X, Bercovy M. "Forgotten knee" after total knee replacement: a pragmatic study from a single-centre cohort. Joint, bone, spine : revue du rhumatisme 2015. doi: 10.1016/j.jbspin.2014.11.006. PubMed PMID: 25623519. 10.1016/j.jbspin.2014.11.006 25623519

[14] Hossain FS, Konan S, Patel S, Rodriguez-Merchan EC, Haddad FS. The assessment of outcome after total knee arthroplasty: are we there yet? The bone & joint journal 2015;97-B(1):3–9. doi: 10.1302/0301-620X.97B1.34434. PubMed PMID: 25568406. 10.1302/0301-620X.97B1.34434 25568406

[15] Giesinger K, Hamilton DF, Jost B, Holzner B, Giesinger JM. Comparative responsiveness of outcome measures for total knee arthroplasty. Osteoarthr Cartil 2014;22(2):184–189. doi: 10.1016/j.joca.2013.11.001. PubMed PMID: 24262431; PubMed Central PMCID: PMC3988962. 10.1016/j.joca.2013.11.001 PMC3988962 24262431

[16] Loibl M, Baumlein M, Massen F, Gueorguiev B, Glaab R, Perren T, et al. Sports activity after surgical treatment of intra-articular tibial plateau fractures in skiers. Am J Sports Med 2013;41(6):1340–1347. doi: 10.1177/0363546513489524. PubMed PMID: 23733831. 10.1177/0363546513489524 23733831

[17] Filbay SR, Ackerman IN, Russell TG, Macri EM, Crossley KM. Health-related quality of life after anterior cruciate ligament reconstruction: a systematic review. Am J Sports Med 2014;42(5):1247–1255. doi: 10.1177/0363546513512774. PubMed PMID: 24318609. 10.1177/0363546513512774 24318609

[18] Tegner Y, Lysholm J. Rating systems in the evaluation of knee ligament injuries. Clin Orthop Relat Res 1985(198):43–49. PubMed PMID: 4028566. 4028566

[19] Lysholm J, Gillquist J. Evaluation of knee ligament surgery results with special emphasis on use of a scoring scale. Am J Sports Med 1982;10(3):150–154. PubMed PMID: 6896798. 10.1177/036354658201000306 6896798

[20] Dawson J, Fitzpatrick R, Murray D, Carr A. Questionnaire on the perceptions of patients about total knee replacement. The Journal of bone and joint surgery British volume 1998;80(1):63–69. PubMed PMID: 9460955. 10.1302/0301-620x.80b1.7859 9460955

[21] Bellamy N, Buchanan WW, Goldsmith CH, Campbell J, Stitt LW. Validation study of WOMAC: a health status instrument for measuring clinically important patient relevant outcomes to antirheumatic drug therapy in patients with osteoarthritis of the hip or knee. J Rheumatol 1988;15(12):1833–1840. PubMed PMID: 3068365. 3068365

[22] Insall JN, Dorr LD, Scott RD, Scott WN. Rationale of the knee society clinical rating system. Clin Orthop Relat Res 1989(248):13–14. PubMed PMID: 2805470. 2805470

[23] Behrend H, Zdravkovic V, Giesinger JM, Giesinger K. Joint awareness after ACL reconstruction: patient-reported outcomes measured with the forgotten joint Score-12. Knee surgery, sports traumatology, arthroscopy : official journal of the ESSKA. 2016. doi: 10.1007/s00167-016-4357-x. PubMed PMID: 27761622. 10.1007/s00167-016-4357-x 27761622

[24] Mokkink LB, Terwee CB, Patrick DL, Alonso J, Stratford PW, Knol DL, et al. The COSMIN checklist for assessing the methodological quality of studies on measurement properties of health status measurement instruments: an international Delphi study. Quality of life research : an international journal of quality of life aspects of treatment, care and rehabilitation 2010;19(4):539–549. doi: 10.1007/s11136-010-9606-8. PubMed PMID: 20169472; PubMed Central PMCID: PMC2852520. 10.1007/s11136-010-9606-8 PMC2852520 20169472

[25] Terwee CB, Bot SD, de Boer MR, van der Windt DA, Knol DL, Dekker J, et al. Quality criteria were proposed for measurement properties of health status questionnaires. J Clin Epidemiol 2007;60(1):34–42. doi: 10.1016/j.jclinepi.2006.03.012. PubMed PMID: 17161752. 10.1016/j.jclinepi.2006.03.012 17161752

[26] Thienpont E, Vanden Berghe A, Schwab PE, Forthomme JP, Cornu O. Joint awareness in osteoarthritis of the hip and knee evaluated with the 'Forgotten Joint' score before and after joint replacement. Knee surgery, sports traumatology, arthroscopy : official journal of the ESSKA. 2016;24(10):3346–3351. doi: 10.1007/s00167-015-3970-4. PubMed PMID: 26740088. 10.1007/s00167-015-3970-4 26740088

[27] Thomsen MG, Latifi R, Kallemose T, Barfod KW, Husted H, Troelsen A. Good validity and reliability of the forgotten joint score in evaluating the outcome of total knee arthroplasty. Acta Orthop 2016;87(3):280–285. doi: 10.3109/17453674.2016.1156934. PubMed PMID: 26937689; PubMed Central PMCID: PMC4900097. 10.3109/17453674.2016.1156934 PMC4900097 26937689

[28] Shadid MB, Vinken NS, Marting LN, Wolterbeek N. The Dutch version of the forgotten joint score: test-retesting reliability and validation. Acta Orthop Belg 2016;82(1):112–118. PubMed PMID: 26984663. 26984663

[29] Matsumoto M, Baba T, Homma Y, Kobayashi H, Ochi H, Yuasa T, et al. Validation study of the forgotten joint Score-12 as a universal patient-reported outcome measure. European journal of orthopaedic surgery & traumatology : orthopedie traumatologie 2015;25(7):1141–1145. doi: 10.1007/s00590-015-1660-z. PubMed PMID: 26148699. 10.1007/s00590-015-1660-z 26148699

[30] Baumann F, Ernstberger T, Loibl M, Zeman F, Nerlich M, Tibesku C. Validation of the German forgotten joint score (G-FJS) according to the COSMIN checklist: does a reduction in joint awareness indicate clinical improvement after arthroplasty of the knee? Arch Orthop Trauma Surg 2016;136(2):257–264. doi: 10.1007/s00402-015-2372-x. PubMed PMID: 26646846. 10.1007/s00402-015-2372-x 26646846

[31] Wirth B, Liffert F, de Bruin ED. [Development and evaluation of a German version of the Lysholm score for measuring outcome after anterior cruciate ligament injuries]. Sportverletzung Sportschaden : Organ der Gesellschaft fur Orthopadisch-Traumatologische Sportmedizin 2011;25(1):37–43. doi: 10.1055/s-0029-1245825. PubMed PMID: 21400391. 10.1055/s-0029-1245825 21400391

[32] Swanenburg J, Koch PP, Meier N, Wirth B. Function and activity in patients with knee arthroplasty: validity and reliability of a German version of the Lysholm score and the Tegner activity scale. Swiss Med Wkly 2014;144:w13976. doi: 10.4414/smw.2014.13976. PubMed PMID: 24921654. 10.4414/smw.2014.13976 24921654

[33] Rabin R, de Charro F. EQ-5D: a measure of health status from the EuroQol group. Ann Med 2001;33(5):337–343. PubMed PMID: 11491192. 10.3109/07853890109002087 11491192

[34] Kellgren JH, Lawrence JS. Radiological assessment of osteo-arthrosis. Ann Rheum Dis 1957;16(4):494–502. PubMed PMID: 13498604; PubMed Central PMCID: PMC1006995. 10.1136/ard.16.4.494 PMC1006995 13498604

[35] Mokkink LB, Terwee CB, Patrick DL (2012). Alonso J.

[36] Luyten FP, Denti M, Filardo G, Kon E, Engebretsen L. Definition and classification of early osteoarthritis of the knee. Knee surgery, sports traumatology, arthroscopy : official journal of the ESSKA. 2012;20(3):401–406. doi: 10.1007/s00167-011-1743-2. PubMed PMID: 22068268. 10.1007/s00167-011-1743-2 22068268

[37] Angele P, Niemeyer P, Steinwachs M, Filardo G, Gomoll AH, Kon E, et al. Chondral and osteochondral operative treatment in early osteoarthritis. Knee surgery, sports traumatology, arthroscopy : official journal of the ESSKA 2016;24(6):1743–1752. doi: 10.1007/s00167-016-4047-8. PubMed PMID: 26922057. 10.1007/s00167-016-4047-8 26922057

[38] Brown EC, 3rd, Clarke HD, Scuderi GR. The painful total knee arthroplasty: diagnosis and management. Orthopedics 2006;29(2):129–136; quiz 37-8. PubMed PMID: 16485456. 10.3928/01477447-20060201-14 16485456

[39] Kraus TM, Martetschlager F, Muller D, Braun KF, Ahrens P, Siebenlist S, et al. Return to sports activity after tibial plateau fractures: 89 cases with minimum 24-month follow-up. Am J Sports Med 2012;40(12):2845–2852. doi: 10.1177/0363546512462564. PubMed PMID: 23118120. 10.1177/0363546512462564 23118120

[40] Felson DT, Niu J, Guermazi A, Sack B, Aliabadi P. Defining radiographic incidence and progression of knee osteoarthritis: suggested modifications of the Kellgren and Lawrence scale. Ann Rheum Dis 2011;70(11):1884–1886. doi: 10.1136/ard.2011.155119. PubMed PMID: 21908453; PubMed Central PMCID: PMC3653624. 10.1136/ard.2011.155119 PMC3653624 21908453

[41] Muraki S, Akune T, Oka H, En-yo Y, Yoshida M, Saika A, et al. Association of radiographic and symptomatic knee osteoarthritis with health-related quality of life in a population-based cohort study in Japan: the ROAD study. Osteoarthr Cartil 2010;18(9):1227–1234. doi: 10.1016/j.joca.2010.06.001. PubMed PMID: 20633679. 10.1016/j.joca.2010.06.001 20633679

